# Moderated Hypofractionated Online Adaptive Radiotherapy in Locally Advanced Cervical Cancer: A Case Report

**DOI:** 10.7759/cureus.66552

**Published:** 2024-08-10

**Authors:** Zheng Zeng, Fuquan Zhang, Junfang Yan

**Affiliations:** 1 Department of Radiation Oncology, Peking Union Medical College Hospital, Chinese Academy of Medical Sciences & Peking Union Medical College, Beijing, CHN

**Keywords:** clinical trial, iterative cone beam computed tomography, online adaptive radiation therapy, moderately hypofractionated radiotherapy, cervical cancer

## Abstract

Cervical cancer is one of the most frequent malignant tumors in females. Concurrent chemoradiotherapy is one of the treatment options for cervical cancer. The treatment time of conventional radiotherapy is long. Moderately hypofractionated radiotherapy (MHRT) offers the advantage of shortening the overall treatment duration and enhancing the radiobiological effects on tumors. MHRT shortens the overall treatment duration while enhancing the radiobiological effects on tumors. Previous studies have reported that MHRT of cervical cancer has relatively high toxicity. Daily online adaptive radiation therapy (oART) showed improvements in dosimetry and a decrease in toxicity. To the best of our knowledge, this case was the first reported case of moderated hypofractionated oART used in a cervical cancer patient to date in a prospective clinical trial (NCT05994300). This case serves as a critical reminder that cervical cancer is a potential tumor that may be in MHRT with iterative cone beam computed tomography-guided oART. Further data are needed to confirm the toxicity and efficacy of this technique.

## Introduction

Cervical cancer ranks as the fourth most prevalent malignancy among women globally, with over 85% of cases occurring in developing countries [1]. Due to the lack of regular screening, more than half of cervical cancer patients are diagnosed with locally advanced cervical cancer (LACC) in some developing countries [2]. Combined external-beam radiotherapy (EBRT) and brachytherapy with chemotherapy is the treatment of choice in LACC [3]. The total overall radiotherapy time usually lasts eight weeks or more [4]. However, many countries with high incidences of cervical cancer have the most limited access to radiotherapy, and the longtime of treatment has a significant impact on radiotherapy access.

The development of three-dimensional planning, intensity-modulated radiation therapy (IMRT), and image guidance have been some significant technological progress in radiation therapy made over the past 20 years [5]. These advancements have enabled greater precision in treatment and reduced the number of radiotherapy fractions needed for various disease sites [6]. At present, moderately hypofractionated radiotherapy (MHRT) has become the standard treatment for prostate cancer and breast cancer [7,8]. In a single-arm prospective study, 50 patients with stage IB-IIIC1 cervical cancers were treated with EBRT 40 Gy in 16 fractions. Three-year disease-free survival rate and overall survival rate were 92.7% and 90.6%, respectively. Acute grade 3 gastrointestinal toxicity and grade 3 genitourinary toxicity were observed in 10 patients (20%) and three patients (6%), respectively [9]. Therefore, MHRT for cervical cancer is expected to achieve similar efficacy to conventional fractionated radiotherapy, but the current techniques have high toxicity that requires further improvement.

Variations in bladder and rectum filling, along with tumor regression, greatly impact the shape and position of the cervix and uterus [10]. As a result, a large margin is used to guarantee sufficient target volume coverage during radiotherapy [10]. A large planning margin would result in excessive irradiation to normal tissue, causing a higher rate of adverse events [11]. At present, iterative cone beam computed tomography (iCBCT)-guided online adaptive radiation therapy (oART) can decrease the margin and irradiated volume compared to IMRT by accommodating per-fractional variations [12]. Previous studies have demonstrated the dosimetric advantages of reduced margins, resulting in a lower incidence of acute toxicity in cervical cancer patients [13].

Therefore, moderated hypofractionated oART may be a promising treatment in LACC. To the best of our knowledge, moderated hypofractionated iCBCT-guided oART was first implemented for cervical cancer, a practice not previously documented in other studies.

## Case presentation

Patient clinical information

A 39-year-old woman was admitted to our hospital for contact vaginal bleeding. She had a histologically confirmed diagnosis of human papillomavirus (HPV) type 16 moderately differentiated positive squamous cell carcinoma of the cervix. A gynecological physical examination revealed the vulva developed normally. The vagina appeared smooth. The cervix exhibited an ulcer-like neoplasm measuring approximately 3 cm in diameter. The uterus was positioned anteriorly and no tenderness. On the left side, there was no extending up to the pelvic wall, while no obvious abnormalities were found on the right side. Routine blood test results, liver function, kidney function, and electrolyte levels were all within normal limits. However, the serum level of squamous cell carcinoma antigen (SCC-Ag) was elevated to 3.3 IU/mL (normal range: 0-2.7 IU/mL), while both carcinoma antigen 125 and carcinoembryonic antigen levels remained normal. The pelvic magnetic resonance imaging (MRI) examination showed a malignant 2.9 x 3.5 x 3.2 cm lesion in the cervix involving left parametria and a left pelvic lymph node 1.4 x 1.5 cm. Positron emission tomography-computed tomography demonstrated a malignant cervical lesion and left pelvic lymph node metastasis, and no distant metastasis was found. According to the International Federation of Gynecology and Obstetrics Staging System, version 2018, the tumor was staged as IIIC1r. According to the American Joint Committee on Cancer TNM Staging System, version 8, it was staged as T2bN1M0 (Stage IIIC1). The case was then discussed in a gynecological multidisciplinary team meeting. The patient was recommended treatment in a phase 1 clinical trial (NCT05994300), and written consent was obtained.

Clinical trial details

NCT05994300 is a prospective, single-institution, phase 1 clinical trial evaluating the clinical efficacy and toxicity of MHRT for patients with biopsy-proven cervical cancer by iCBCT-guided oART. The enrolled patients include patients with stages IB1-IIB and IIIC1 disease, excluding the largest node size is more than 1.5 cm, more than three metastatic lymph nodes, and metastatic lymph nodes located in the common iliac chain. EBRT was performed on the Ethos system (Varian Medical Systems, Palo Alto, CA) in all patients, which is our institution’s iCBCT-guided oART unit. The primary outcome of this study is acute toxicity at three months. The primary outcomes of this study are late toxicity, response evaluation, disease-free survival, overall survival, and quality of life.

Statistical analysis

Statistical analyses were conducted using IBM SPSS Statistics for Windows (version 23.0; IBM Corp, Armonk, NY). Two-sided P-values ≤ 0.05 were considered statistically significant. The mean, median, and standard deviation of the data were determined. If the data were normally distributed, a paired t-test was utilized for statistical analysis; alternatively, the Wilcoxon rank sum test was applied when normality assumptions were not met.

Treatment

**Figure 1:**
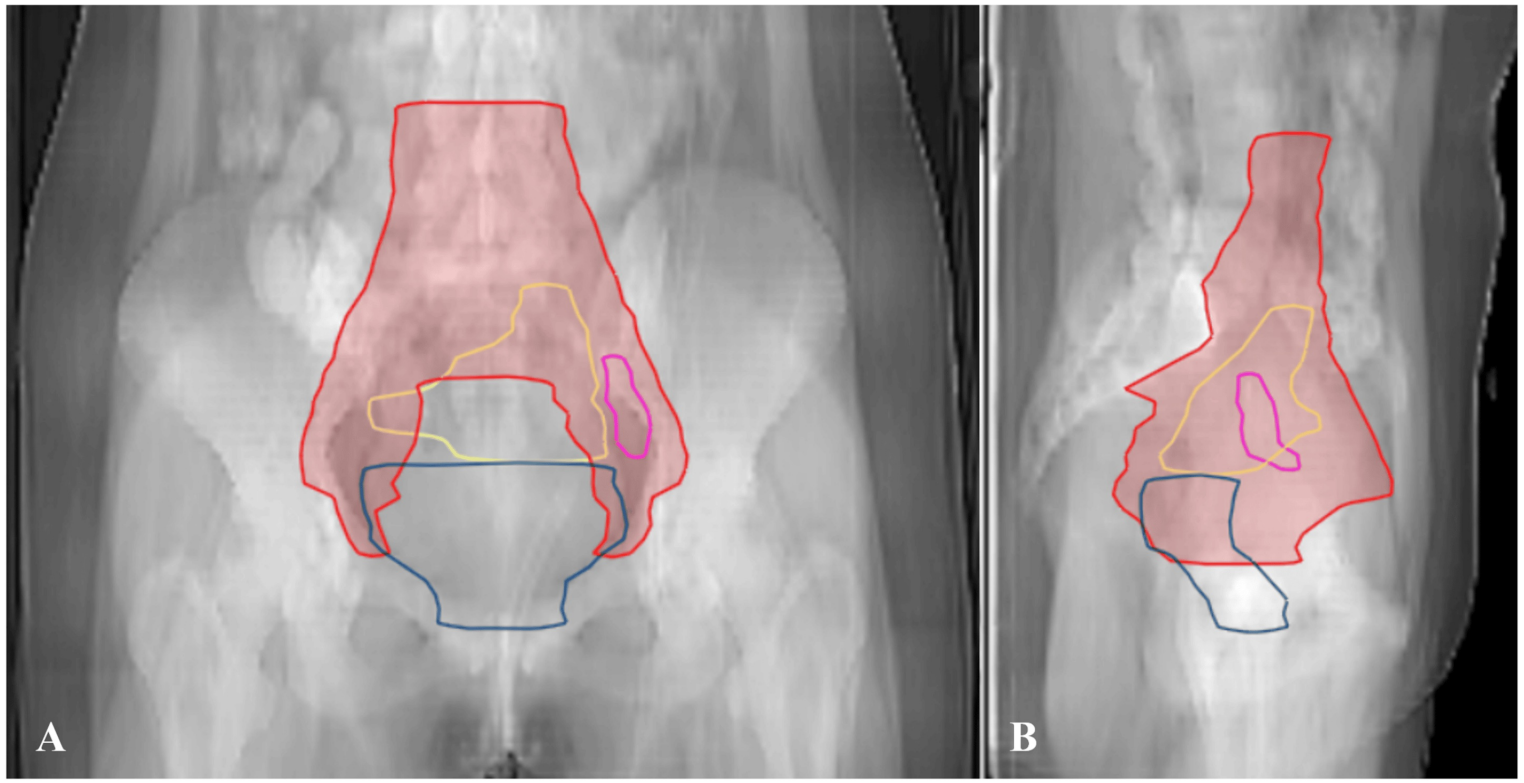
Example images of axial (A) and sagittal (B) slices of a planned CT scan from the patient. CTV-N is shown in red, CTV-U is shown in yellow, CTV-C is shown in blue, and GTVnd is shown in magenta. Abbreviations: CT, computed tomography; CTV-N, clinical target volume of the lymph; CTV-U, clinical target volume of the uterus; CTV-C, clinical target volume of the cervix; GTVnd, gross tumor volume node

This patient received concurrent chemoradiotherapy (CCRT), in which radiotherapy consisted of EBRT with daily iCBCT-guided oART and brachytherapy. The clinical target volume (CTV) contouring was performed according to the Radiation Therapy Oncology Group Consensus Guidelines [14]. CTV of the lymph (CTV-N) encompassed the pelvic lymphatic drainage area, CTV of the uterus (CTV-U) encompassed the uterus, and CTV of the cervix (CTV-C) included the cervix, vagina, and adjacent parametrial regions [15]. Gross tumor volume nodes (GTVnd) included metastatic lymph nodes (Figure 1). A uniform three-dimensional planning margin of 3 mm GTVnd to plan the target volume node (PTV-nd), 5 mm CTV to plan the target volume of the lymph (PTV-N), and to plan the target volume of the cervix (PTV-C) margin was used for CTV-C and CTV-N, and a 10 mm margin was used CTV-U to the generated planned target volume of the uterus (PTV-U). The prescribed dose consisted of 43.55 Gy in 17 fractions and a simultaneous integrated boost of 54.40 Gy in 17 fractions to GTVnd. Dose constraints for critical organs at risk (OARs) were as follows: bladder D50% ≤ 3800 cGy, rectum D50% ≤ 4000 cGy, bowel D2 cc ≤ 4550 cGy, bowel D50% ≤ 1800 cGy, and spinal cord D0.1cc ≤ 4000 cGy, respectively. Nine-field IMRT plans were created using the Ethos treatment planning system.

During the treatment with oART, the patient received 17 fractions of oART and cooperated fully with the entire adaptive procedure. This included the initial acquisition of iCBCT; influencer generation and editing, as well as the generation and editing of targets and OARs; and finally plan generation and selection. The timing data are shown in Table 1. The adaptive procedure averaged 18 minutes and 34 seconds. After incorporating a second iCBCT scan and treatment, the total average time increased to 23 minutes and 34 seconds.

**Table 1:** Timing data of online adaptive radiation therapy. Abbreviations: iCBCT, iterative cone beam computed tomography; OARs, organs at risk

Times (minutes and seconds) consuming	Min	Max	AVG
Initial acquisition of iCBCT	48 s	59s	52S
Influencer generation and edits	1 min 01 s	3min21s	2min11s
Target and OARs generation and edits	5 min 25 s	11min16s	7min40s
Plan generation and selection	3 min 03 s	6min43s	3min51s
Second acquisition of iCBCT	45 s	57s	51s
Treatment	4 min 10 s	4min56s	4min09s
Online adaptive time	10 min 17 s	22min12s	18min34s
Total time	15 min 12 s	28min12s	23min34s

**Figure 2:**
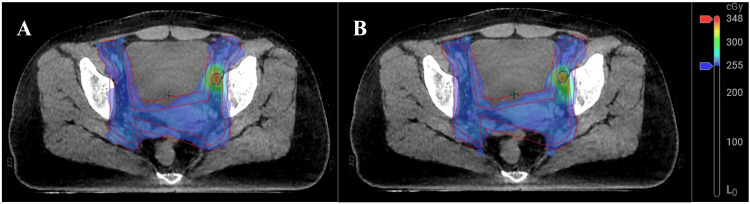
Comparison of the adapted plan and scheduled plan using iterative cone beam computed tomography-guided oART for this patient with LACC. Adapted plan (A) and scheduled plan (B) for fraction two of treatment. Abbreviations: LACC, locally advanced cervical cancer; oART, online adaptive radiation therapy

For treatment plan selection, the adapted plan was chosen for all 17 fractions. The adapted plan achieved superior dosimetric coverage of the target volume compared to the scheduled plan (Figure 2). The median V100% values for CTV-N, CTV-C, CTV-U, GTVnd, PTV-N, PTV-C, PTV-U, and PTV-nd were 99.9% versus 99.4% (P = 0.009), 98.4% versus 92.5% (P < 0.001), 99.1% versus 97.8% (P = 0.002), 100.0% versus 99.8% (P = 0.029), 96.9% versus 97.9% (P = 0.357), 93.2% versus 87.6% (P < 0.001), 96.5% versus 93.8% (P < 0.001), and 98.5% versus 96.3% (P = 0.005), respectively.

Table 2 presents the dosimetric results of OARs across all treatment fractions for the patient. In the adaptive plan, the mean dose and D2cc of bowel were 100.0 ± 15.2 cGy and 266.9 ± 0.8 cGy, respectively, which were significantly lower than the doses in the scheduled plan (142.6 ± 29.9 cGy and 268.3 ± 1.2 cGy, respectively). In contrast to the scheduled plan, the adaptive plan demonstrated a significant improvement in rectal dosimetry at V4000 cGy (P < 0.001). However, there was no significant difference in the mean dose and V4000 cGy of the bladder. The dosimetry of the bone marrow was also improved with the adaptive plan.

**Table 2:** Comparison of the dosimetric outcomes of OARs between the adapted plan and the scheduled plan for the patients. Abbreviations: OARs, organs at risk

OARs	Goal	Scheduled plan	Adapted plan	P
Femur head left	D5%(cGy)	143.4±5.7	139.7±9.8	0.202
Femur head right	D5%(cGy)	144.3±5.7	136.4±21.3	0.322
Bone marrow	D90%(cGy)	48.4±2.0	37.8±2.8	＜0.001
Bladder	Dmean (cGy)	146.8±21.7	156.6±21.1	0.207
	V4000 cGy(%)	24.6±9.6	21.6±7.6	0.768
Rectum	Dmean (cGy)	176.9±18.4	163.5±21.8	0.048
	V4000 cGy (%)	31.2±6.4	18.6±9.1	＜0.001
Bowel	Dmean (cGy)	142.6±29.9	100.0±15.2	＜0.001
	D2cc (cGy)	268.3±1.2	266.9±0.8	＜0.001

**Figure 3:**
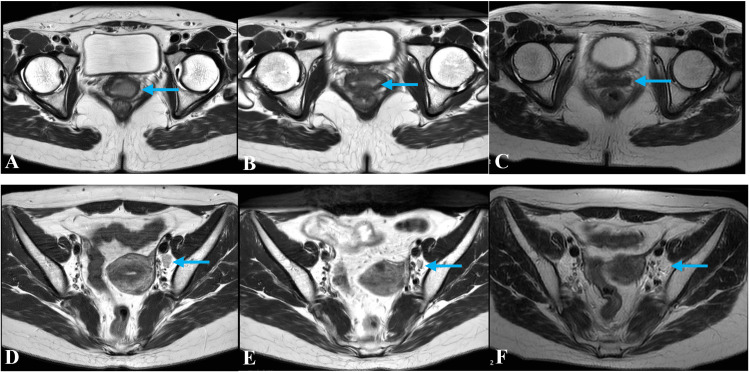
Pelvic MRI images of the patient. A, D: Pelvic MRI T2 images of the patient before treatment. B, E: Pelvic MRI T2 images of the patient after EBRT. C, F: Pelvic MRI images of the patient one month after treatment. MRI: magnetic resonance imaging; EBRT: external-beam radiotherapy

After the completion of oART, treatment response was evaluated on the basis of the findings of pelvic MRI in accordance with RECIST 1.1 (https://recist.eortc.org/recist-1-1-2/) (Figure 3). The total diameter of the measurable lesion was about 1.1 × 1.8 × 1.2 cm, which is reduced by 56% from the baseline. The total diameter of the target lymph node was about 0.6 × 0.4 cm, which is reduced by 52% from the baseline. The serum level of SCC-Ag was decreased to 2.2 IU/mL (0-2.7 IU/mL). CT-guided high-dose-rate (HDR) brachytherapy generally began after EBRT, and a dose of 30 in five fractions was delivered. Intravenous cisplatin was administered weekly at a dosage of 40 mg/m^2^ during radiotherapy. Following this, the patient received five cycles of treatment with cisplatin. Moderated hypofractionated oART with HDR brachytherapy appears to be a well-tolerated treatment, and only grade 2 acute hematologic toxicity and grade 1 acute gastrointestinal toxicity were observed. No acute genitourinary toxicity was observed. Pelvic MRI showed that clinical response after one month of CCRT. The overall follow-up time was 10 months, and no local recurrence or distant metastasis happened.

## Discussion

Here, we presented an LACC patient with stage IIIC, and the size of the tumor was relatively large. After completing treatment with moderated hypofractionated oART, the tumor size decreased significantly. Then, HDR brachytherapy was implemented, and the patient ultimately achieved a clinical response. No grade 3 and more acute toxicity was reported. The overall follow-up time was 10 months, and this patient achieved a continuous recurrence-free survival. For all we know, this is the first reported case of LACC treated with moderated hypofractionated oART.

Radiotherapy is essential in the treatment of cervical cancer, with around 80% of patients receiving radiation therapy either alone or in combination with other treatments [5]. Radiotherapy for cervical cancer includes both EBRT and brachytherapy, which usually lasts eight weeks [4]. However, prolonged treatment duration can lead to insufficient availability of medical resources [6]. MHRT offers the advantage of shortening the overall treatment duration while enhancing the radiobiological effects on tumors [7]. MHRT has been used and become the standard of care in different cancer sites and offers radiobiologic superiority for tissues with a low α/β ratio [7,8]. However, the radiobiological effect of MHRT in cervical cancer remains unknown.

Several studies have reported the outcomes of MHRT for cervical cancer. Muckaden et al. [16] conducted a retrospective analysis of 62 stage IIIB cervical cancer patients who received pelvic irradiation at 39 Gy in 13 fractions combined with brachytherapy. The five-year disease-free survival rate was 59%, with five patients experiencing late grade 3 rectal toxicity [16]. Kavuma et al. [17] showed the treatment outcomes of definitive CCRT utilizing an MHRT regimen of 45 Gy in 15 fractions with two-dimensional radiotherapy, noting that 10%-15% of patients experienced late gastrointestinal toxic effects of grade 3 or higher. More recently, a phase II randomized trial from Iran has compared MHRT of 40 Gy in 15 daily fractions with weekly cisplatin using three-dimensional conformal radiotherapy with the standard treatment of 45 Gy in 25 daily fractions with weekly cisplatin [17]. The results indicated that 66.7% of patients in the standard group and 65.5% in the MHRT group achieved a complete clinical response (P = 0.13). However, the MHRT group experienced a significantly higher incidence of acute grade ≥3 gastrointestinal toxicity (27.6%) compared to the standard group (6.7%) (P = 0.032).

Currently, an ongoing Thai phase II trial is randomizing patients with locally advanced cervical cancer between EBRT with 44 Gy/20 fractions or 45 Gy/25 fractions. EBRT is being delivered with IMRT and weekly concurrent cisplatin. The authors reported grade 2 and more acute gastrointestinal toxicity (50%) and genitourinary toxicity (28%) in the MHRT arm [18]. From two-dimensional radiotherapy to IMRT, the experience of a clinical series of cervical cancer patients treated with MHRT may bring a higher incidence of adverse reactions.

Adaptive radiotherapy, a promising technique for treating cervical cancer, still has many areas that need further exploration. These include margin determination, dosimetry, and toxicity [19]. The previous study found that oART could reduce planning target volume margins to 5 mm in the postoperative treatment of endometrial and cervical cancer, which significantly decreases the dose of critical OARs. Wang et al. [13] evaluated further the clinical implementation of daily iCBCT-guided oART in the postoperative treatment of endometrial and cervical cancer with planning target volume margin reduction to 5 mm [20]. Daily oART also showed improvements in dosimetry. Meanwhile, grade 3 toxicities were observed in only one patient with leukopenia, and no patients experienced acute urinary toxicity. In short, oART has proven beneficial in treating cervical cancer safely.

MHRT is relatively high toxicity; therefore, demonstrating the feasibility of MHRT to cervical cancer using oART is critical to increasing the accessibility of this technique to patients with cervical cancer.

## Conclusions

Herein, we described the first reported case of a patient with cervical cancer treated with 43.35 Gy in 17 fractions of CTV and 54.40 Gy in 17 fractions of GTV-nd using oART. Moderated hypofractionated oART with HDR brachytherapy appears to be a very promising treatment in LACC. Further long-term follow-up of this patient and others treated in phase 1 clinical trial (NCT05994300) is needed to assess the duration of response.
